# Ambipolar organic phototransistors based on 6,6′-dibromoindigo†

**DOI:** 10.1039/c8ra02346h

**Published:** 2018-04-19

**Authors:** Hyoeun Kim, Gyoungsik Kim, Inho Song, Jungho Lee, Hanum Abdullah, Changduk Yang, Joon Hak Oh

**Affiliations:** Department of Chemical Engineering, Pohang University of Science and Technology (POSTECH) Pohang Gyeongbuk 790-784 South Korea joonhoh@postech.ac.kr; Department of Energy Engineering, School of Energy and Chemical Engineering, Perovtronics Research Center, Low Dimensional Carbon Materials Center, Ulsan National Institute of Science and Technology (UNIST) 50 UNIST-gil, Ulju-gun Ulsan 44919 South Korea yang@unist.ac.kr

## Abstract

Ambipolar organic phototransistors were fabricated using a natural pigment 6,6′-dibromoindigo (6-BrIG) as the active channel. These phototransistors yielded significantly enhanced currents upon light illumination with photoresponsivities and external quantum efficiencies as high as 10.3 A W^−1^ and 2437% for the n-channel, and 55.4 mA W^−1^ and 13.1% for the p-channel, respectively. In addition, simple inverter complementary circuits were fabricated by integrating two ambipolar phototransistors. Channel current was dependent on light intensity and voltage bias. This study provides a basis for an in-depth understanding of the optoelectronic characteristics of 6-BrIG, and introduces this material as an ecofriendly candidate for optoelectronic applications.

## Abstract 2

Ambipolar phototransistors with a natural pigment, 6,6′-dibromoindigo (6-BrIG or tyrian purple), have been investigated.

## Graphical abstract

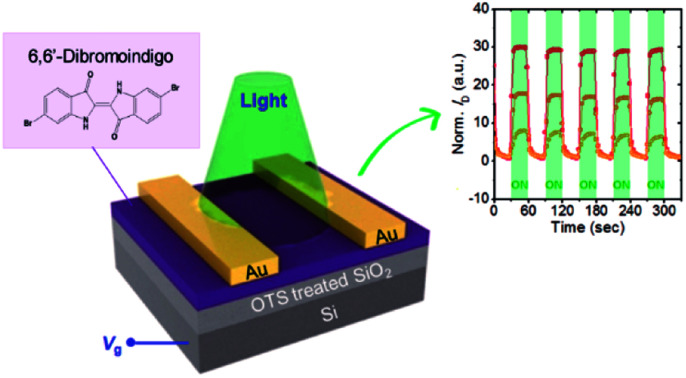

## 1. Introduction

Organic π-conjugated materials have received much attention due to their tunable optical and electrical properties, suitability for flexible device fabrication, and large-area processability.^1–3^ Organic field-effect transistors (OFETs),^4,5^ organic light-emitting diodes (OLEDs),^6^ organic phototransistors (OPTs),^7–9^ organic photovoltaics (OPVs),^10,11^ organic memories,^12^ and organic sensors^13^ have been successfully developed. Phototransistors, which allow one to control channel conductance *via* light absorption, are particularly applicable to electronic devices such as light sensors and optical switches.^14^ Although many research groups have reported phototransistors with unipolar characteristics,^15–19^ few have explored ambipolar phototransistors, which have mostly been demonstrated using blend,^20^ heterojunction,^21,22^ or complementary logic circuits^23^ made by sequentially processing two p- and n-type semiconductors. Phototransistors based on ambipolar materials, which are single component systems that can function as both p- and n-type semiconductors, have also been demonstrated.^24–27^ Single component, ambipolar transistors are cost-effective and boast several advantages including large-area applicability and simple fabrication. Despite their advantages, research on ambipolar material-based phototransistors is insufficient. Therefore, new photoconductive ambipolar materials must be developed to improve our understanding of their photophysical properties and suitability for practical applications.

In this work, we investigated the use of a natural pigment, 6,6′-dibromoindigo (6-BrIG or tyrian purple), as an ambipolar semiconductor for use in phototransistors. 6-BrIG is a well-known chromophore and is the oldest known purple dye, extracted from sea snails. Interestingly, this material has several unique properties that match well with the requirements of electronic devices.^28–30^6-BrIG boasts (i) a small bandgap energy of 1.8 eV for balanced ambipolar charge injection, (ii) a relatively deep-lying lowest unoccupied molecular orbital (LUMO) level of −3.7 eV for air-stable electron transport, (iii) strong intra- and intermolecular hydrogen bonding between its amine and carbonyl groups; this induces planarity and short π–π stacking distances, which are beneficial for efficient charge transport, and (iv) non-toxicity and biocompatibility.^29^ Although ambipolar OFETs and inverter circuits based on 6-BrIG have been demonstrated,^31–33^ early studies have been confined to its use in transistors.

The small bandgap and broad absorption band (350–700 nm) of 6-BrIG imply facile excitation from the highest occupied molecular orbital (HOMO) to the LUMO upon exposure to visible light. We therefore introduced 6-BrIG into phototransistors as an ambipolar organic semiconductor. Ambipolar charge transport was observed in the dark and was significantly enhanced upon exposure to light. This indicated that 6-BrIG functioned as a photoactive layer. OPTs based on 6-BrIG exhibited photoresponsivity as high as 10.3 A W^−1^ and remarkable external quantum efficiency of 2437% under light illumination.

## 2. Experimental

### 2.1 Instruments

6-BrIG was synthesized in accordance with previously published procedures.^34,35^^1^H NMR spectra were recorded on a Varian VNRS 400 MHz (Varian USA) spectrophotometer using CDCl_3_ as the solvent and tetramethylsilane (TMS) as the internal standard. UV-vis spectra were acquired on a Cary 5000 spectrophotometer. Cyclic voltammetry (CV) was performed on an AMETEK VersaSTAT 3 with a three-electrode cell in a nitrogen-purged solution of 0.1 M tetra-*n*-butylammonium hexafluorophosphate (*n*-Bu_4_NPF_6_) in acetonitrile at a scan rate of 0.1 V s^−1^ at room temperature. A platinum wire, Ag/Ag^+^ (0.01 M of AgNO_3_ in acetonitrile) electrode, and 6-BrIG deposited onto ITO-coated glass were used as the counter electrode, reference electrode, and working electrode, respectively. The Ag/Ag^+^ reference electrode was calibrated using a ferrocene/ferrocenium redox couple as an external standard, whose oxidation potential was set at −4.8 eV with respect to the zero vacuum level. The HOMO energy level was obtained from the equation HOMO = −(*E*^onset^_ox_ − E^onset^_ferrocene_ + 4.8) eV. The LUMO level was obtained from the equation LUMO = HOMO + *E*^opt^_g_ eV.

### 2.2 Synthesis of 6-bromo-3-iodoindole (1)

Iodine (2.59 g, 10.2 mmol) was added to a solution of 6-bromoindole (2 g, 10.2 mmol) and sodium hydroxide (0.41 g, 10.2 mmol) in methanol (100 mL) and an aqueous potassium iodide (1.69 g, 10.2 mmol) solution (10 mL). The reaction mixture was stirred at room temperature for 3 h and water was added. The resulting precipitate was collected by filtration, washed with water, and dried under reduced pressure. The product 6-bromo-3-iodoindole was used for the following reaction without purification.

### 2.3 Synthesis of 3-acetoxy-6-bromoindole (2)

Silver acetate (1.56 g, 9.32 mmol) was added to a solution of 6-bromo-3-iodoindole (2 g, 6.21 mmol) in glacial acetic acid (50 mL). The reaction mixture was stirred at 90 °C for 2 h and the mixture was cooled to room temperature and filtered. The crude product was purified by column chromatography (silica gel, dichloromethane) to afford 0.88 g (56%) of 3-acetoxy-6-bromoindole. ^1^H NMR (400 MHz, CDCl_3_) *δ* (ppm) 7.89 (br, 1H), 7.47 (d, 1H, *J* = 1.6 Hz), 7.42 (d, 1H, *J* = 8.4 Hz), 7.32 (d, 1H, *J* = 2.8 Hz), 7.25 (dd, 1H, *J*_1_ = 8.4 Hz, *J*_2_ = 1.6 Hz), 2.36 (s, 3H). GC-DIP/MS (*m*/*z*) calcd: 254.08; found: 254.7. EA: anal. calcd for C_10_H_8_BrNO_2_: C, 47.27; H, 3.17; N, 5.51; found: C, 47.11; H, 3.31; N, 5.37.

### 2.4 Synthesis of 6,6′-dibromoindigo (6-BrIG)

1 M sodium hydroxide solution (10 mL) was added to a solution of 3-acetoxy-6-bromoindole (0.88 g, 3.47 mmol) in ethanol (30 mL). Water was added to the mixture after stirring at room temperature for 3 h. The resulting precipitate was collected by filtration and washed several times with water and cold ethanol. The resulting purple solid was dried under vacuum and combined to give a purple solid as 6,6′-dibromoindigo (0.55 g, 75%). NMR peaks were not detected due to limited solubility in various solvents. IR *ν*_max_ (solid)/cm^−1^: 3385 (N–H), 1633 (C=O), 1610 (C=C). GC-DIP/MS (*m*/*z*) calcd: 420.05; found: 419.9. EA: anal. calcd for C_16_H_8_Br_2_N_2_O_2_: C, 45.75; H, 1.92; N, 6.67; found: C, 45.50; H, 1.71; N, 6.51.

### 2.5 Device fabrication

Devices were fabricated with a bottom-gate top-contact configuration. Commercially available, thermally grown, 300 nm-thick SiO_2_ on highly n-doped Si (100) (<0.004 Ω cm) was used as the device substrate. Si functioned as both the substrate and the gate electrode, and SiO_2_ acted as the gate dielectric with a capacitance per unit area of 10 nF cm^−2^. The SiO_2_ surface was modified with an *n*-octadecyltrimethoxysilane (OTS) self-assembled monolayer according to a reported method.^36^ The OTS-treated wafers were rinsed with toluene, acetone and isopropyl alcohol, and dried under a stream of N_2_. For the channel layer, 6-BrIG was deposited by thermal evaporation under high vacuum (<5.0 × 10^−6^ torr, *T*_substrate_ = 80 °C, rate = 0.1–0.3 Å s^−1^, and film thickness = 30 nm). Thermal annealing was carried out at 100 °C for 30 min in an N_2_ atmosphere. Au top-contact electrodes (40 nm) were thermally evaporated through a shadow mask with a channel width–length ratio (*W*/*L*) of 17.5.

### 2.6 Measurement of OFETs and OPTs

Electrical performance was recorded in a N_2_-filled glovebox using a Keithley 4200 semiconductor parametric analyzer due to their low air-stability. Optoelectronic properties were measured in a vacuum chamber using monochromic and polychromic light sources.

## 3. Results and discussion

### 3.1 Synthesis and characterization

**Scheme 1:**
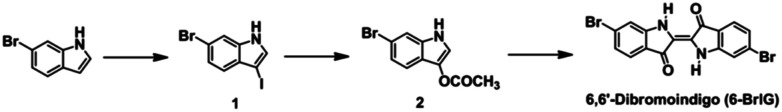
Synthetic routes to 6-BrIG.

The synthetic procedures to obtain intermediates and the target molecule (6-BrIG) are outlined in Scheme 1, which were prepared according to the literatures.^34,35^ Briefly, 3-acetoxy-6-bromoindole (2) was synthesized by sequential reactions, *i.e.*, iodination of 6-bromoindole and nucleophilic substitution with silver acetate, followed by hydrolysis with a sodium hydroxide solution in ethanol, affording 6-BrIG in a 25.7% overall yield. Detailed synthetic routes and characterization can be found in the Experimental section and ESI.† The synthesized 6-BrIG showed limited solubility in common organic solvents, mainly due to strong intramolecular hydrogen bonding. Thus, 6-BrIG was difficult to characterize by NMR. Therefore, the structural characteristics and elemental composition of the synthesized 6-BrIG were verified by mass spectrometry. Both were consistent with calculated values. In addition, the infrared absorption spectrum of 6-BrIG contained stretching vibrations at 3385, 1633, and 1610 cm^−1^, corresponding to N–H, C=O, and C=C bonds, respectively. Thermogravimetric analyses revealed a high thermal stability with a decomposition temperature of 346 °C, which corresponded to a 5% weight loss (see Fig. S1 and S2 in ESI†).

**Fig. 1:**
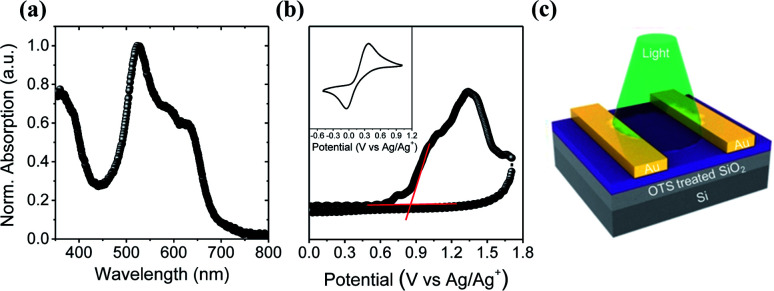
(a) UV-vis absorption spectrum of 6-BrIG film. (b) Cyclic voltammogram of 6-BrIG film evaporated on ITO. (c) Schematic device structure of the phototransistor.

The UV-vis absorption spectrum of a 6-BrIG thin film is shown in Fig. 1a and the corresponding spectral data are summarized in Table 1. Interestingly, despite its small structure, 6-BrIG exhibited a broad absorption band from 350–700 nm, which can be attributed to a combination of the H-chromophore and a complex set of intramolecular interactions induced by the indigo core,^37–39^ and an absorption maximum (*λ*_max_) at 523 nm. The estimated optical bandgap (*E*^opt^_g_) of 6-BrIG was 1.82 eV.

**Table 1:** UV-vis absorption and electrochemical properties of the 6-BrIG

	*λ* ^film^_max_ (nm)	*E* ^opt^_g_ a (eV)	*E*_HOMO_ b (eV)	*E*_LUMO_ c (eV)
6-BrIG	523	1.82	−5.49	−3.67

a Calculated from the absorption band edge of the thin film, *E*^opt^_g_ = 1240/*λ*_edge_.

b Vacuum deposited thin film in 0.1 M CH_3_CN/*n*-Bu_4_NPF_6_, *versus* ferrocene/ferrocenium at 0.1 V s^−1^. Energy level of HOMO estimated from the onset oxidation potentials, assuming the absolute energy level of ferrocene/ferrocenium to be 4.8 eV below vacuum.

c *E*_LUMO_ (eV) = *E*_HOMO_ + *E*^opt^_g_.

Cyclic voltammetry (CV) was conducted with a working electrode consisting of thermally evaporated 6-BrIG on indium tin oxide (ITO)-coated glass. An *n*-Bu_4_NPF_6_ solution was used as the electrolyte and ferrocene was employed as an external standard (Fig. 1b). The HOMO level was determined as the first onset potential of the oxidation peak at 0.86 V, but no reduction peak was observed in the potential range. The HOMO level of 6-BrIG, calculated using the equation *E*_HOMO_ = −(*E*^onset^_ox_ − *E*^onset^_ferrocene_ + 4.8) eV, was −5.49 eV and the LUMO level, calculated with *E*^opt^_g_, was −3.67 eV. These energy levels are commensurate with ambipolar charge transport in OFETs.

#### Characterizations of the thin films

A schematic device structure with a bottom-gate top-contact configuration is shown in Fig. 1c. 6-BrIG thin films were prepared by thermal evaporation onto OTS-pretreated SiO_2_/Si substrates; the SiO_2_ layer thickness was 300 nm. Film morphology and the thickness of the semiconducting layer are key factors in the optimization of phototransistor devices. Well-packed microstructural grains are important for efficient charge transport, while the thickness of the semiconducting layer is vital for sufficient light absorption. If the film is too thick, light may not be able to penetrate the nanometer-thin channel due to internal filtering. Light is absorbed into the non-accumulated bulk semiconductor, which acts as an optical filter, before reaching the channel.^40–42^ Conversely, if the semiconducting layer is too thin, grains will not be sufficiently connected and will not absorb sufficient light. In our work, 6-BrIG film thickness was optimal at 30 nm and the films were annealed at 100 °C.

**Fig. 2:**
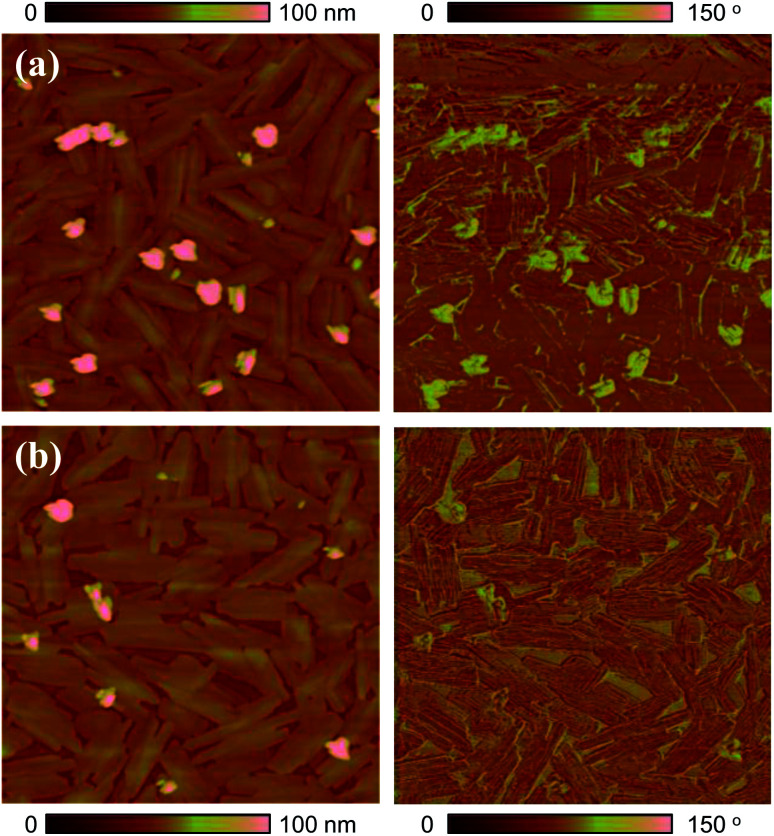
Tapping-mode AFM height (left) and phase (right) images (5 μm × 5 μm) of 6-BrIG films on OTS-treated SiO_2_/Si substrates: (a) as-cast and (b) annealed film at 100 °C.

The surface morphology of 6-BrIG films was investigated *via* tapping-mode atomic force microscopy (AFM) before and after annealing at 100 °C for 30 min (Fig. 2). Thin films of 6-BrIG consisted of large, crystalline, needle-like grains with an average length of 1.16 μm. After annealing, a relatively more uniform and flatter surface was formed, with a root-mean-square (rms) roughness of 8.56 nm, much lower than that of the as-cast film (13.3 nm). This result indicates an enhancement of intermolecular packing and charge transport after the thermal annealing.

#### OPT performances

**Fig. 3:**
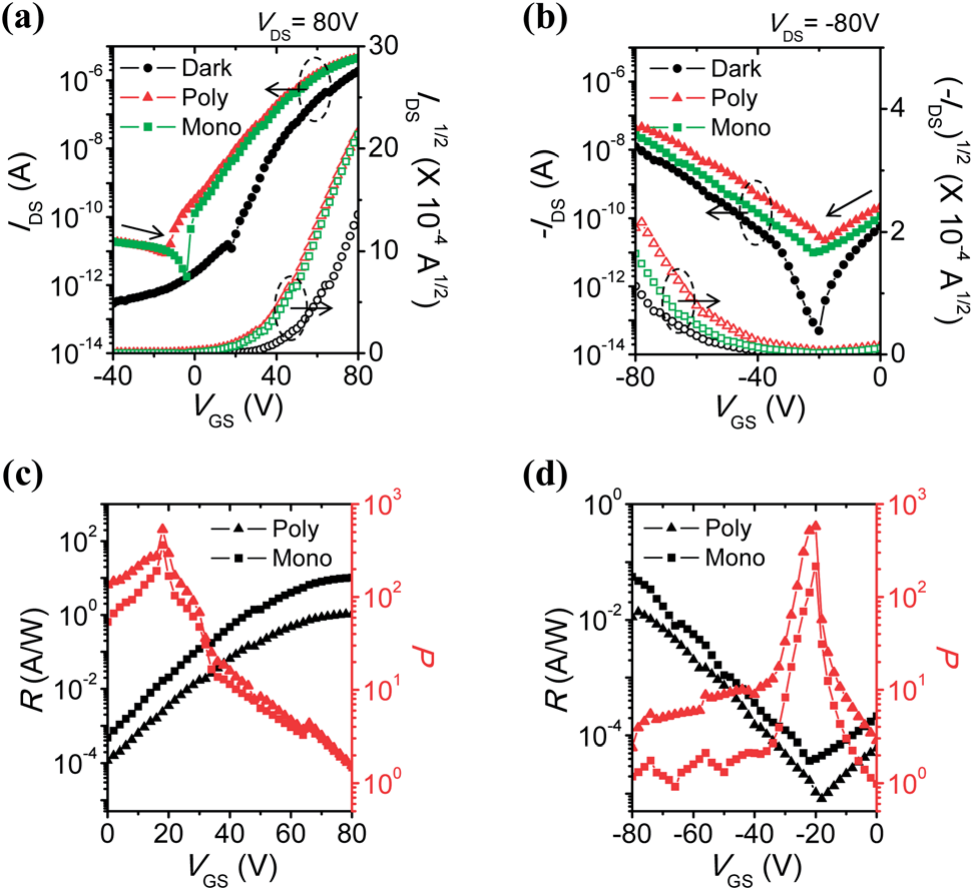
Transfer characteristics, photoresponsivity (*R*), and photocurrent/dark-current ratio (*P*) of 6-BrIG film in the dark and under illumination: (a and c) n-dominant operation, sweep from −80 to 80 V and (b and d) p-dominant operation, sweep from 80 to −80 V. The circle, triangle, and square symbols represent the dark, polychromic, and green monochromic light, respectively.

Fig. 3a, b and S3† show the *I*_DS_–*V*_GS_ curves measured in the dark and under illumination at *V*_DS_ = 80 V and −80 V, respectively. Photoelectronic properties were investigated using green monochromic (*λ* = 523 nm, *P*_green_ = 530 μW cm^−2^) and white polychromic (*P*_white_ = 5.4 mW cm^−2^) light sources, respectively. Typical ambipolar performance with dominant electron transport was observed from 6-BrIG; OFET characteristics in the saturation region were extracted using the following eqn (1):

1 *I*_DS_ = 1/2(*W*/*L*)*μC*_i_(*V*_GS_ − *V*_TH_)^2^

where *W* and *L* are the channel width and length, respectively, *μ* is the field-effect mobility, *C*_i_ is the capacitance per unit area of the dielectric layer, and *V*_TH_ is the threshold voltage. The electrical characteristics in the dark and under illumination are summarized in Table S1.†

Under light irradiation, off-currents significantly increased in both electron- and hole-enhancement modes due to the creation and dissociation of photogenerated excitons. Therefore, the on/off ratios under illumination became smaller, from 10^7^ to 10^5^–10^6^ for n-channel transport, and from 10^5^ to 10^3^ for p-channel transport. In addition, mobilities were enhanced and the threshold voltages shifted negatively in the n-channel (or positively in the p-channel), meaning the easier turn-on of the device. This can be attributed to a decrease in the density of trap sites by photogenerated charge carriers.^43,44^ The amount of electrons (or holes) increases owing to the active charges coming from the additional photogenerated charge carriers, not only from gate-induced effects.

Photoresponsivity (*R*) and the photocurrent/dark-current ratio (*P*) are quantitative parameters for the sensitivity of OPTs. These values were calculated by the following eqn (2) and (3):

2 *R* = (*I*_light_ − *I*_dark_)/*P*_inc_

3 *P* = (*I*_light_ − *I*_dark_)/*I*_dark_

where *I*_light_ is the drain current under illumination, *I*_dark_ is the drain current in the dark, and *P*_inc_ is the incident illumination power on the active channel of the device, respectively. The OPT of 6-BrIG shows a maximum *R* of 10.3 A W^−1^ in the n-channel (0.0554 A W^−1^ in p-channel) and *P* of 532 in the n-channel (574 in p-channel) under green and white light irradiation, respectively (Fig. 3c and d and Table 2). This implies that *R* is closely related to the absorption range of the photoactive material, 6-BrIG (see UV-vis absorption spectra in Fig. 1a), whereas *P* is more dependent on the incident optical power. For example, *P*_white_ (5.4 mW cm^−2^) was ten-fold higher than *P*_green_ (0.53 mW cm^−2^).^15^

**Table 2:** OPT performance of 6-BrIG under light illumination

	n-channel			p-channel		
	*R*_max_ (A W^−1^)	*P*_max_	*η*_max_ (%)	*R*_max_ (A W^−1^)	*P*_max_	*η*_max_ (%)
Mono chromic	10.3	364	2437	0.0554	214	13.1
Poly chromic	1.05	532	—	0.0137	574	—

In addition, the external quantum efficiency (EQE, *η*) of OPTs can be defined as the ratio of the number of photogenerated carriers to the number of incident photons in the OPT channel, calculated as follows:

4 *η* = {(*I*_light_ − *I*_dark_)*hc*}/(*eP*_inc_*λ*_peak_)

where *h* represents Planck's constant, *c* is the speed of light, *e* is the fundamental unit of charge, and *λ*_peak_ is the peak wavelength of incident light, respectively. Only a monochromic light source can be used for these measurements. The n-channel exhibited a high *η*_n_ value up to 2437% at |*V*_GS_| = 80 V under green light irradiation, while the p-channel showed an *η*_p_ of 13% under the same conditions. Thus, the electron photocurrent was much larger than the hole photocurrent, indicating that 6-BrIG is a strongly n-dominant ambipolar material. We also tested thickness-dependent phototransistor performances to find the optimal film thickness. When the film is too thin (15 nm), grains are not sufficiently connected, which precludes device operation (Fig. S4†). On the other hand, when the film is thick (60 nm), light is absorbed into the bulk active layer before reaching the channel, reducing phototransistor performances (Fig. S5†). Therefore, we concluded that the optimized thickness of semiconductor film is around 30 nm in phototransistor applications.

#### Characterization of photocurrent response

**Fig. 4:**
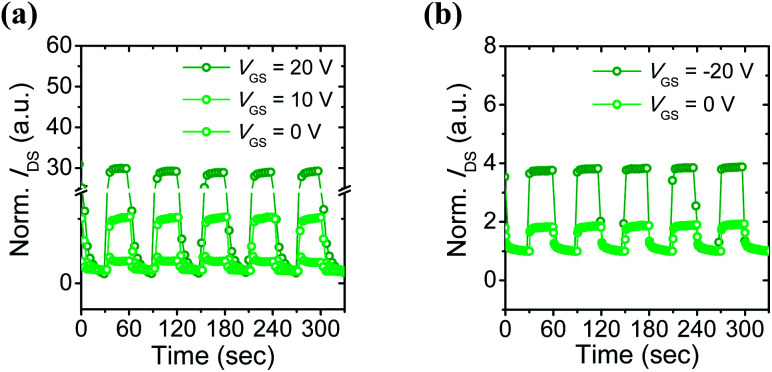
Photocurrent responses of the 6-BrIG film under green light exposure with a time interval of 30 s: (a) n-dominant operation at *V*_DS_ = 20 V and (b) p-dominant operation at *V*_DS_ = −80 V.

To investigate the real-time photocurrent response of 6-BrIG phototransistors, *I*_DS_ was measured as a function of time using a pulsed exposure with green light at time intervals of 30 s, as shown in Fig. 4. The rise and decay times for light-on and -off modes were estimated to be in the range of few seconds (Fig. S6†). In both n- and p-dominant operation, significant increases in photocurrent were observed with increasing *V*_GS_.

In particular, the normalized *I*_DS_ was about 2 at *V*_GS_ = 0 V but increased dramatically to nearly 30 at *V*_GS_ = 20 V in the n-channel. Likewise, a two-fold increase in the normalized *I*_DS_ was obtained by applying a negative bias. These results may be due to amplification effects, which are one of the advantages of a transistor platform.

**Fig. 5:**
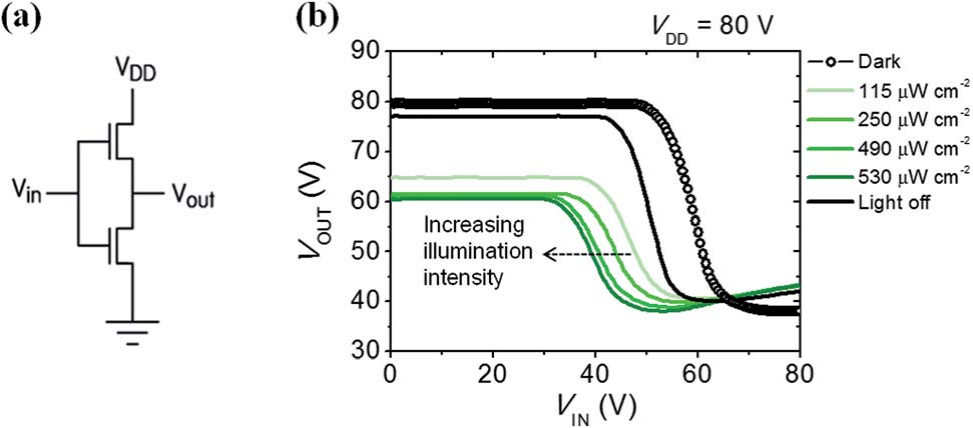
(a) Circuit diagram of the inverter. (b) Voltage transfer (*V*_OUT_–*V*_IN_) characteristic curve measured in the dark and under green light with illumination intensities between 115–530 μW cm^−2^.

Using their ambipolar characteristics of photoconductive semiconductors, complementary metal-oxide-semiconductor (CMOS)-like inverter were fabricated. Voltage transfer curves from the inverter consisting of 6-BrIG are plotted at *V*_DD_ = 80 V and under various light intensities, such as dark, 115, 250, 490, and 530 μW cm^−2^, as shown in Fig. 5. The gain characteristics as a function of *V*_IN_ at different incident optical intensities are also presented in Fig. S7.† The close dependence of inverter characteristics and incident light power can be explained as follows. (i) At low *V*_IN_, *V*_OUT_ decreases with increasing light intensity because photogenerated electrons function as leakage current in n-channel off mode. (ii) In the middle region, where *V*_IN_ is near the n-type threshold voltage, the sharp decrease point shifts from 48 V to 30 V with increasing light power. This is attributed to a greater change in the threshold voltage of the n-channel (43.6 V to 35.8 V) than in the p-channel (−51.7 V to −51.2 V) with or without illumination. (iii) At high *V*_IN_, *V*_OUT_ increases slightly with increasing optical power due to additional leakage from photogenerated holes in the p-channel off mode. These results confirm that the photocurrent of 6-BrIG depends on both voltage bias and light intensity. In such ambipolar phototransistors and complementary circuits, both light illumination and gate bias can be used to modulate the transport of semiconducting channel.^45^

## 4. Conclusions

This report describes the first fabrication and characterization of ambipolar phototransistors based on 6-BrIG. The small optical bandgap of 6-BrIG allowed for excitation with visible light. OFETs made with 6-BrIG exhibited typical ambipolar performance with both high photoresponsivity (*R*_n_ = 10.3 A W^−1^) and excellent external quantum efficiency (*η*_n_ = 2437%) in n-channel mode. However, 6-BrIG is an n-dominant ambipolar material and p-channel mode yielded relatively low figures of merit (*R*_p_ = 0.0554 A W^−1^ and *η*_p_ = 13.1%). In addition, photocurrent response was reversible and variable with voltage bias and light intensity. These findings open new possibilities for the low-cost fabrication of organic optical sensors with tunable polarity and photoresponsivity.

## Conflicts of interest

There are no conflicts to declare.

## Supplementary Material

RA-008-C8RA02346H-s001
