# Supplementary material for: Ambipolar organic phototransistors based on 6,6′-dibromoindigo
Source: RSC Adv. 2018 Apr 19;8(27):14747–52. doi: 10.1039/c8ra02346h (PMC9079942; doi:10.1039/c8ra02346h)
Supplement: RA-008-C8RA02346H-s001 [file RA-008-C8RA02346H-s001.pdf]

Electronic Supplementary Material (ESI) for RSC Advances.
This journal is © The Royal Society of Chemistry 2018

Supporting Information for:

Ambipolar Organic Phototransistors Based on 6,6'-Dibromoindigo

Hyoeun Kim,[a] *Gyoungsik Kim,*[b] *Inho Song,*[a] *Jungho Lee,*[b] *Hanum Abdullah,*[a] *Changduk Yang,*[*,b] *and Joon Hak Oh*[*,a]

[a]Department of Chemical Engineering, Pohang University of Science and Technology (POSTECH), Pohang, Gyeongbuk 790-784, South Korea, E-mail: joonhoh@postech.ac.kr

[b]Department of Energy Engineering, School of Energy and Chemical Engineering, Perovtronics Research Center, Low Dimensional Carbon Materials Center, Ulsan National Institute of Science and Technology (UNIST), 50 UNIST-gil, Ulju-gun, Ulsan 44919, South Korea, E-mail: yang@unist.ac.kr

Supporting Figures

Infrared spectrum for 6-BrIG (Supporting **Figure 1**) -- S3

Thermogravimetric analysis of 6-BrIG (Supporting **Figure 2**) -- S3

Transfer curves of 6-BrIG (Supporting **Figure 3**) -- S4

AFM results of 6-BrIG films with different film thickness (Supporting **Figure 4**) ----------------------------- S4

EQE comparison of 6-BrIG phototransistors with different film thickness (Supporting **Figure 5**) ----------- S5

Switch-on and -off photocurrent responses of 6-BrIG (Supporting **Figure 6**) ---------------------------------- S5

Voltage gain of the complementary inverter (Supporting **Figure 7**) -- S6

Supplementary Tables

OFET performance of 6-BrIG film in the dark and under illumination --
(Supporting **Table 1**) -- S6

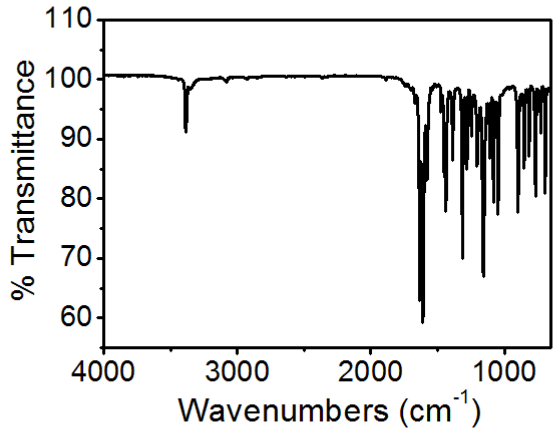

Figure S1. Infrared spectrum for 6-BrIG.

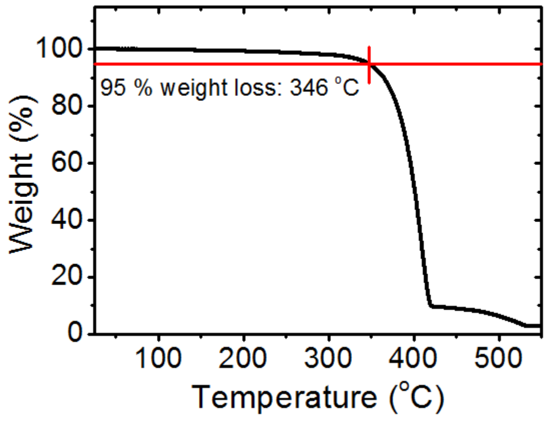

Figure S2. Thermogravimetric analysis of 6-BrIG.

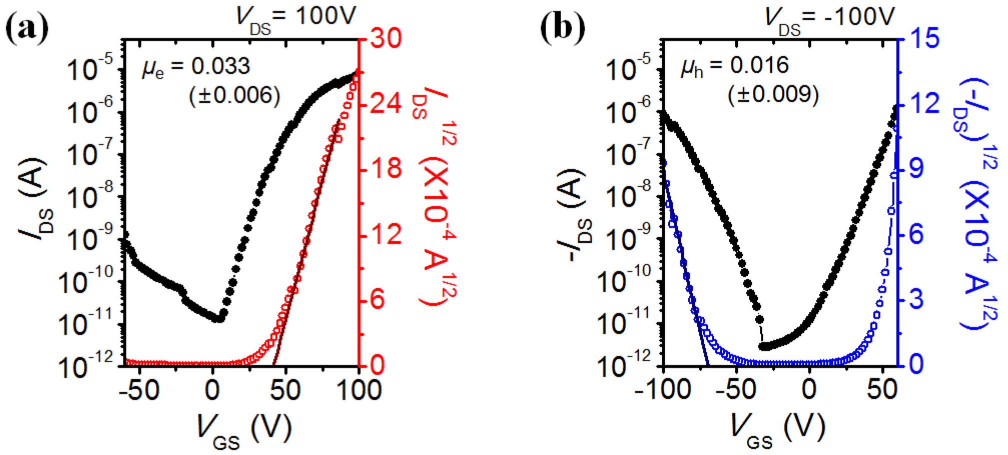

Figure S3. Current-voltage (I_{DS} - V_{GS}) characteristics measured in a N_2-filled glovebox: (a) electron-enhancement operation, V_{DS} = 100 V and (b) hole-enhancement operation, V_{DS} = -100 V.

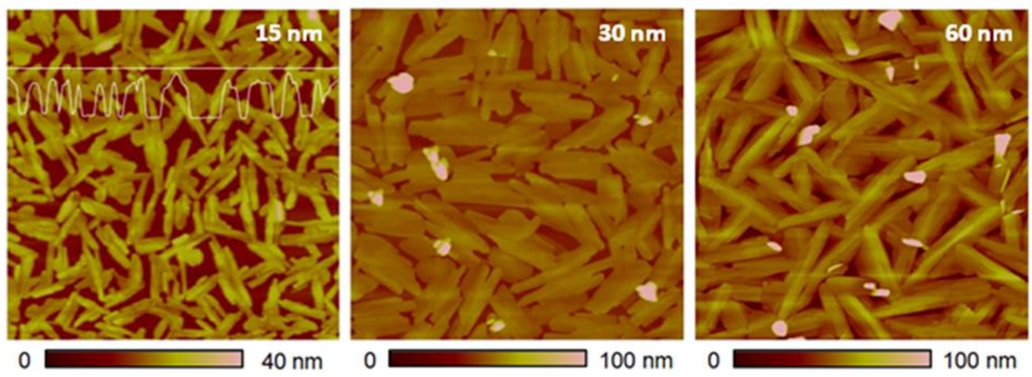

Figure S4. Tapping-mode AFM images (5 μm × 5 μm) of 6-BrIG films with different film thickness on OTS-treated SiO_2/Si substrates annealed at 100℃.

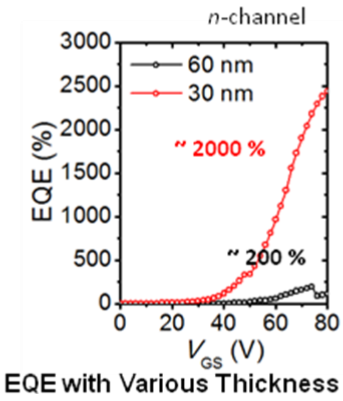

Figure S5. Comparison of the EQE values of 6-BrIG phototransistors with different film thicknesses.

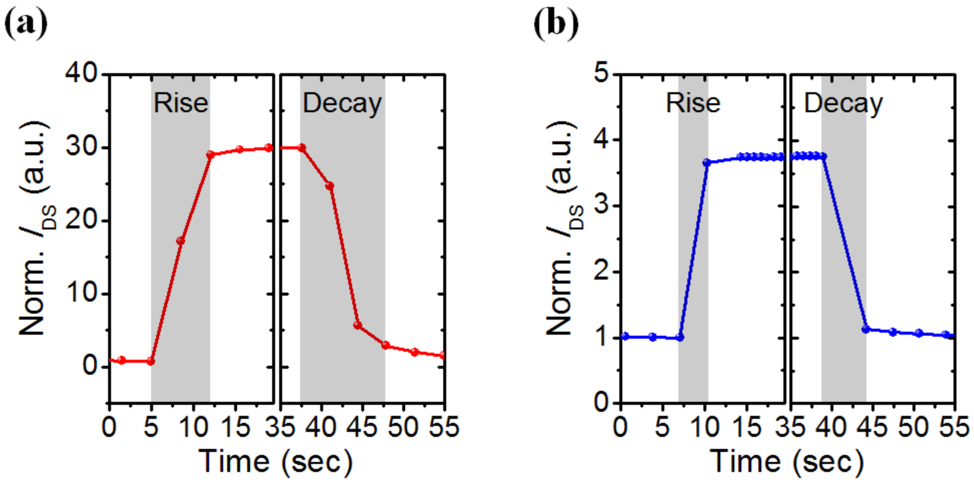

Figure S6. Switch-on and -off photocurrent responses as a function of time at $|V_{GS}|$ = 20 V: (a) *n*-dominant operation applying V_{DS} = 20 V and (b) *p*-dominant operation applying V_{DS} = -80 V.

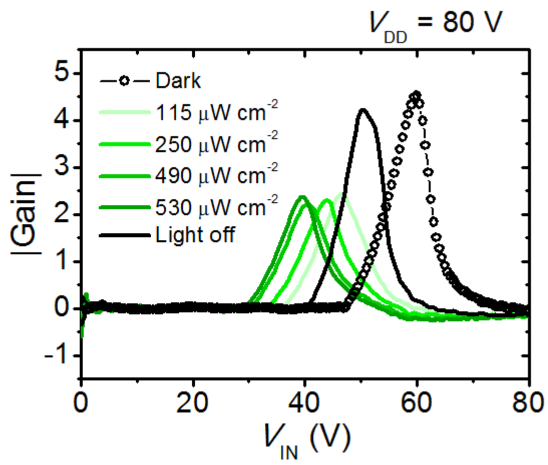

Figure S7. Voltage gain of the complementary inverter in dark and under green light with illumination intensities between 115-530 μW cm^{-2}.

Table S1. OFET performance of 6-BrIG film in the dark and under illumination.

	n-channel			*p*-channel		
	$\mu_{e,max}$[a] (cm^2 V^{-1} s^{-1})	I_{on} / I_{off}	V_{TH} (V)	$\mu_{h,max}$ (cm^2 V^{-1} s^{-1})	I_{on} / I_{off}	V_{TH} (V)
Dark	0.031 (0.030 ± 0.0004)[b]	> 10^7	43.6	5.8×10^{-4} $(4.9 \pm 0.8) \times 10^{-4}$	> 10^5	-51.7
Mono chromic	0.034 (0.032 ± 0.0009)	> 10^6	35.8	7.6×10^{-4} $(6.4 \pm 0.5) \times 10^{-4}$	> 10^3	-51.2
Poly chromic	0.032 (0.031 ± 0.0006)	> 10^5	33.8	8.7×10^{-4} $(7.8 \pm 0.6) \times 10^{-4}$	> 10^3	-47.7

[a] The maximum mobilities of the OFET devices (L = 50 μm and W/L = 17.5). [b] The average mobilities and standard deviations are shown in parenthesis
